# Venolymphatic malformation (VLM) manifestation in the knee: An uncommon encounter

**DOI:** 10.1016/j.radcr.2024.10.105

**Published:** 2024-11-22

**Authors:** Sakshi Dudhe, Devyansh Nimodia, Gaurav V. Mishra, Pratapsingh Hanuman Parihar, Anjali kumari, Rishitha Kotla

**Affiliations:** a Department of Radiodiagnosis, Datta Meghe Institute of Medical Sciences, Sawangi, Wardha, Maharashtra, India 442001; b Department of Psychiatry, Datta Meghe Institute of Medical Sciences, Sawangi, Wardha, Maharashtra, India, 442001

**Keywords:** Case report, MRI, Ultrasound, Slow flow, Venolymphatic malformations

## Abstract

An uncommon congenital disorder known as venolymphatic malformation (VLM) of the knee develops from improper venous and lymphatic system development. It is a specific kind of vascular abnormality that affects lymphatic and veins, causing abnormal growth and dilation of these vessels. These types of malformations do not directly connect to the main channels but instead appear as swelling or other characteristic features depending on the specific type of malformation. Diagnosis can be particularly challenging when the skin covering the area appears normal. We present a 12-year-old male who presented with pain and swelling on the supra-patellar part of right knee for three months. He experienced some painonly during movements. Magnetic resonance imaging (MRI) and ultrasound were used for evaluation. It was confirmed that the patient had a VLM in the vastus medialis, vastus lateralis, and vastus intermedis muscles, intermuscular plane, and suprapatellar bursa region, along with a Morrant Baker cyst. The diagnostic modalities of MRI can be used with confidence to diagnose VLM swellings in the extremities.

## Introduction

VLMs are typically of congenital origin and occur due to the abnormal growth of embryonic vascular tissue. These malformations were previously referred to as either lymphangio-hemangiomas or hemangio- lymphangiomas [1]. Although the specific etiology of VLM is unknown, it is thought to arise when the lymphatic and venous systems do not correctly develop during embryonic development. The outcome is dilated blood vessels that may develop cystic gaps or channels that can contain fluid, blood, or both.

Depending on the extent and location of the deformity, the symptoms of VLM of the knee might vary greatly. While some individuals might not have any symptoms at all, others might have pain, swelling, or discomfort in the affected area. The VLM may occasionally result in deformity or functional disability, such as trouble running or walking.

In order to diagnose VLM of the knee, a combination of clinical examination, imaging tests, and biopsy is usually used. Due to its ability to offer precise information on the location and size of the malformation, MRI is commonly utilized as an imaging modality for VLM. The severity of the condition and the patient's symptoms will determine the best course of treatment for VLM of the knee. In some circumstances, conservative methods like physical therapy or compression garments may be successful in lowering symptoms. If the deformity is causing severe pain or functional impairment, more intrusive therapies like sclerotherapy or surgical excision may be required. Overall, VLM of the knee is a very uncommon disorder that has the potential to significantly impair a person's quality of life and result in considerable disability.

However, many people with VLM of the knee can effectively manage their symptoms and keep up an active, normal lifestyle with early detection and the right therapy.

## Case report

**Fig. 1:**
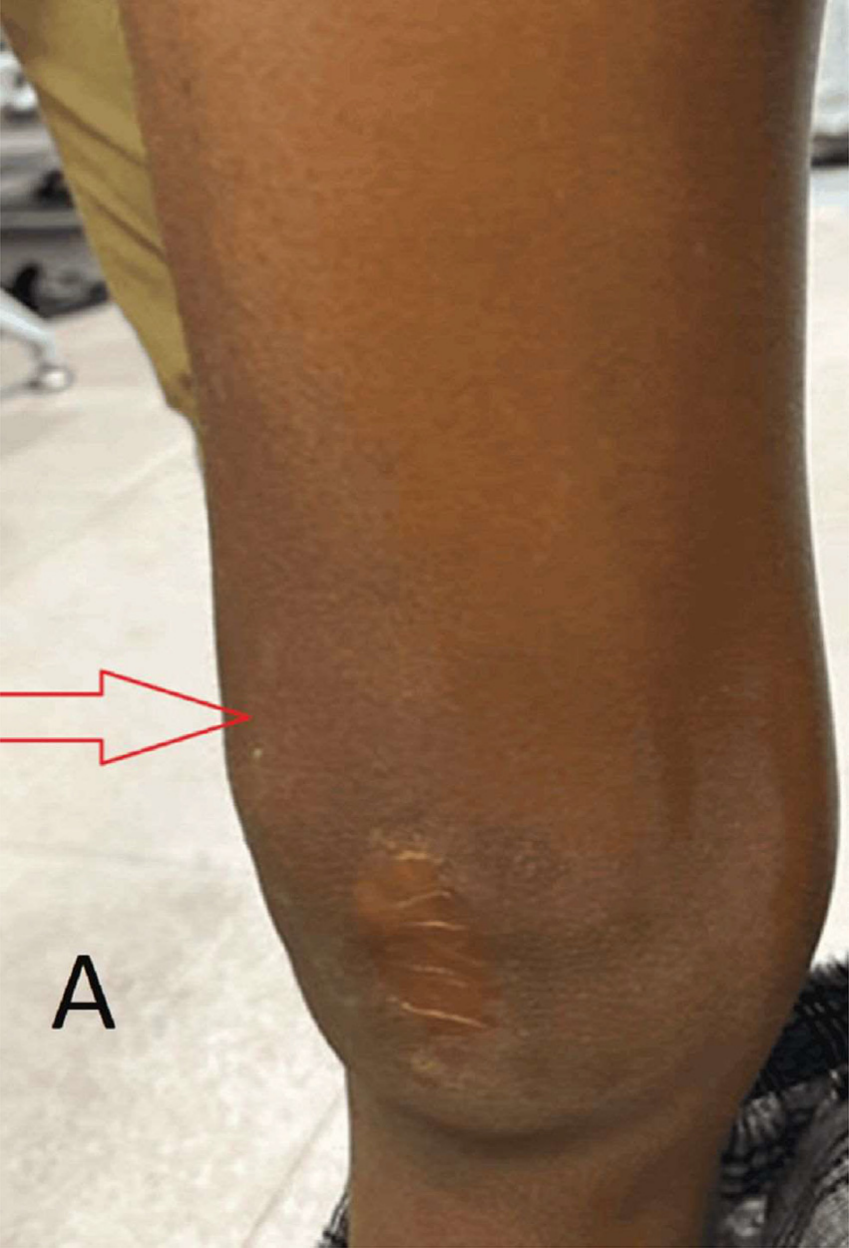
On inspection of right knee, swelling on the supra-patellar aspect of the right knee (red arrow), along with skin discolouration and scarring was noted.

A 12‑year‑old male patient came to our orthopedics outpatient department with swelling and pain over the supra-patellar aspect of right knee for three months. He had history of trauma due to fall two years prior, while playing football. The intensity of pain had increased since last 3 months, prompting him to visit our hospital. He now complained of worsening on and off pain in central part of right knee joint. The patient did not have any significant medical or family history. There was no evidence of any prior systemic illnesses. The patient aggravated with movement of knee joint and during activities such as playing sports or walking long distances. Upon local examination, there was swelling on the supra-patellar aspect of the right knee, measuring 2.5 cm × 3.2 cm, along with skin discoloration and scarring (Fig. 1). Flexion of the affected knee was restricted.

**Fig. 2:**
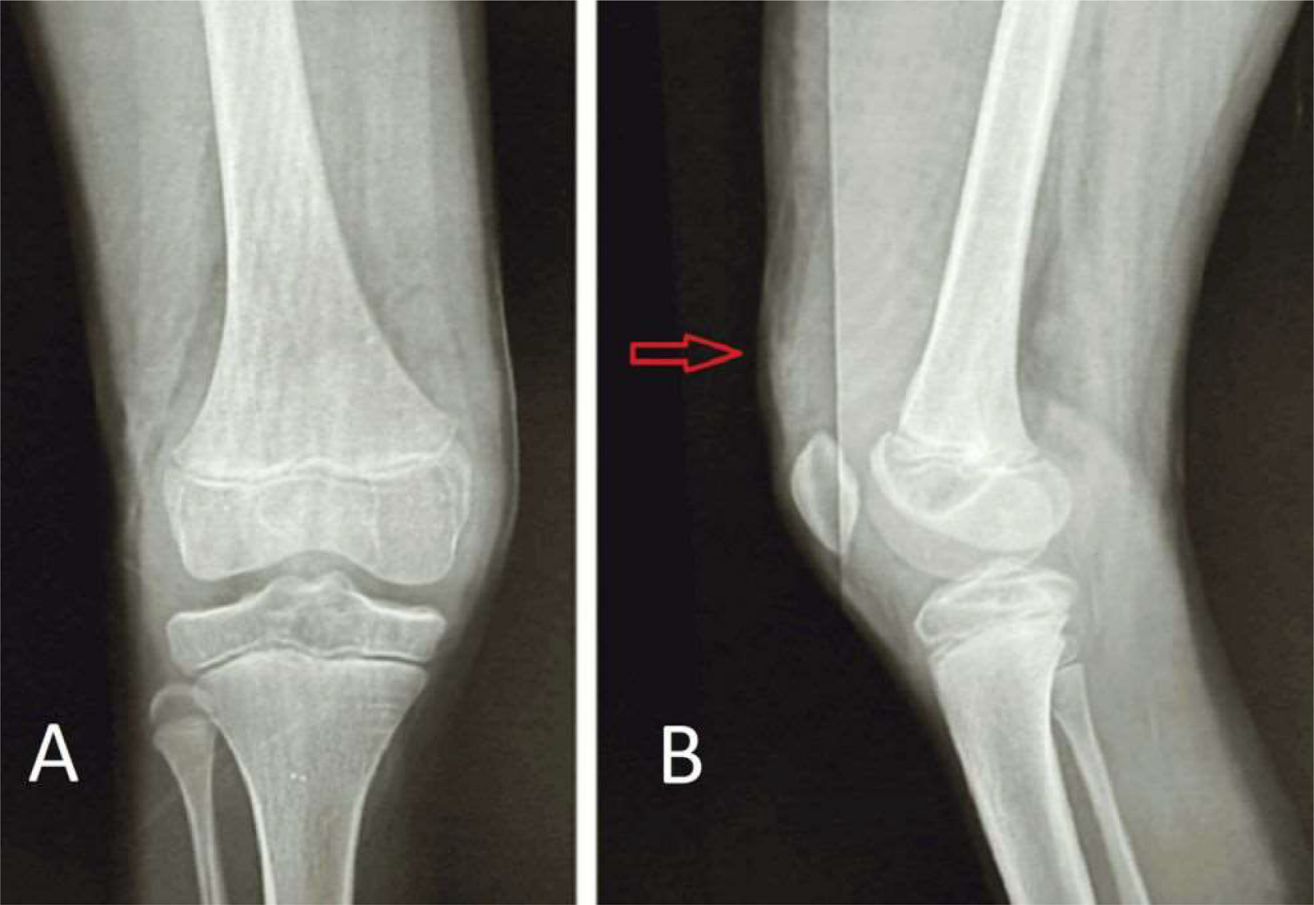
X-ray AP view and Lateral view showing normal knee joint with mild soft tissue swelling on the supra-patellar aspect of right knee (red arrow). A-Xray AP view,B-Xray Lateral view.

**Fig. 3:**
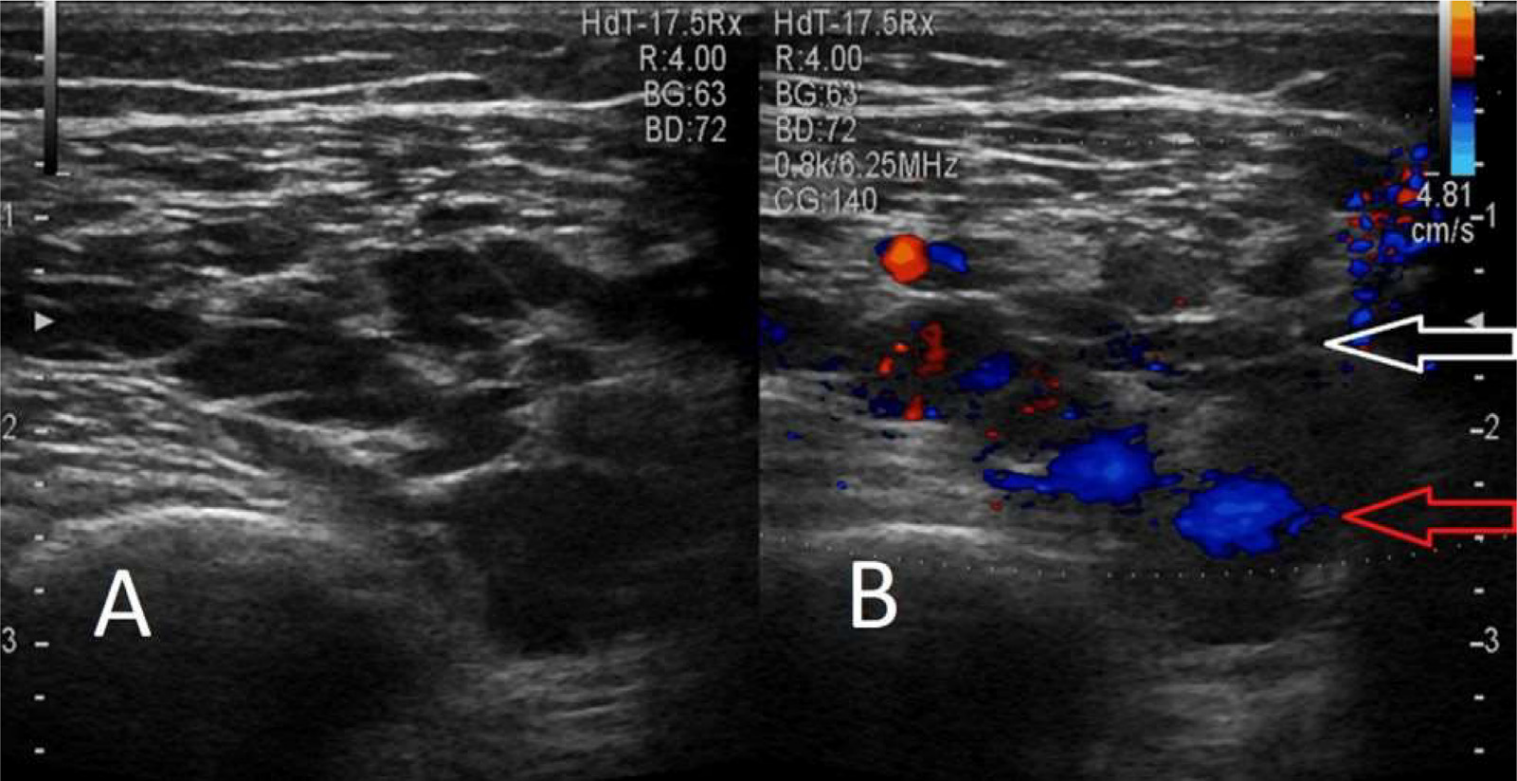
Ultrasound (USG) grey scale B mode image of right knee joint showing multicystic anechoic lesion with few slow-flow vascular channels (white arrow-lymphatic channels, red arrow-venous channels). A-Grey scale B mode image, B-Grey scale B mode image with color doppler.

**Fig. 4:**
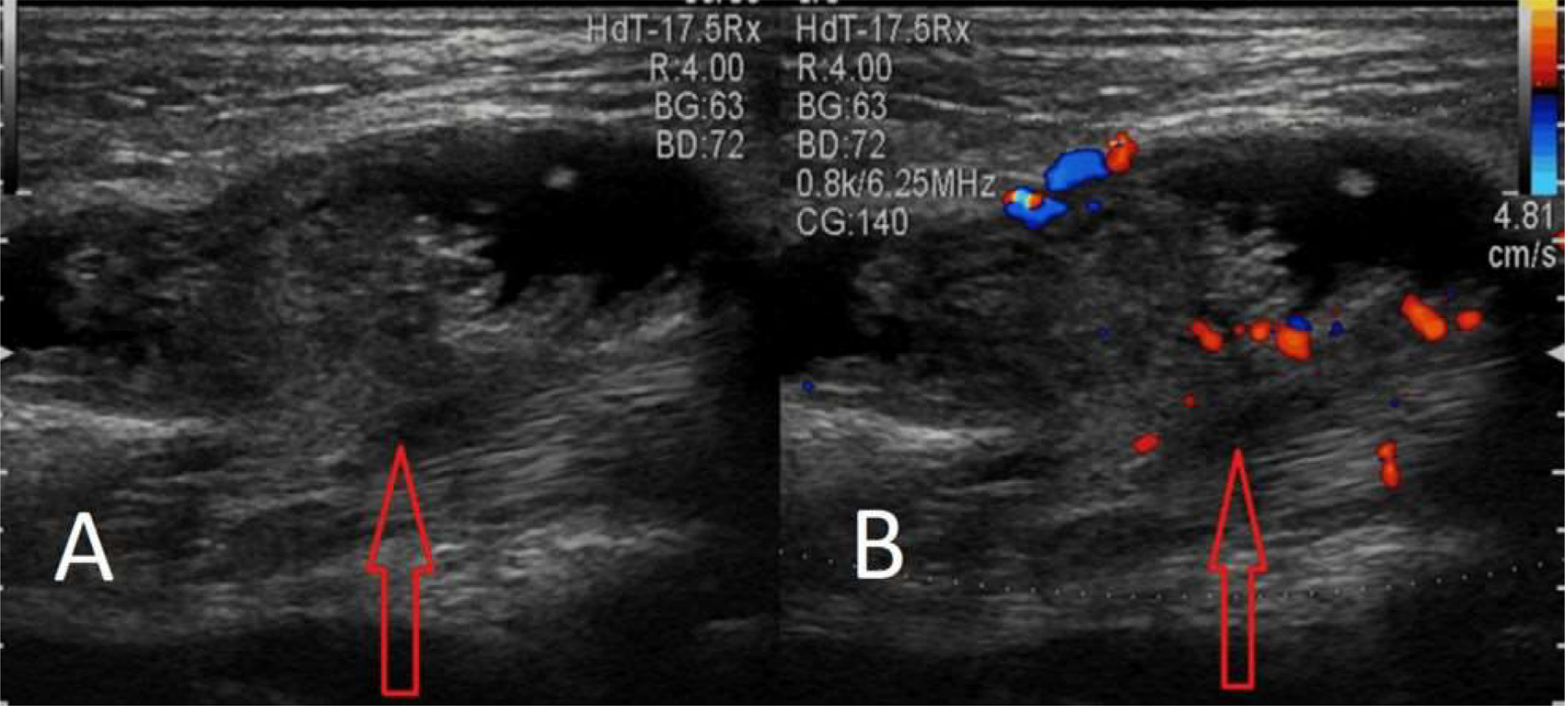
Ultrasound grey scale B mode image of right popliteal fossa showing cystic lesion with internal echoes suggestive of morrant baker cyst (red arrows). A-Grey scale B mode image, B-Grey scale B mode image with color doppler.

**Fig. 5:**
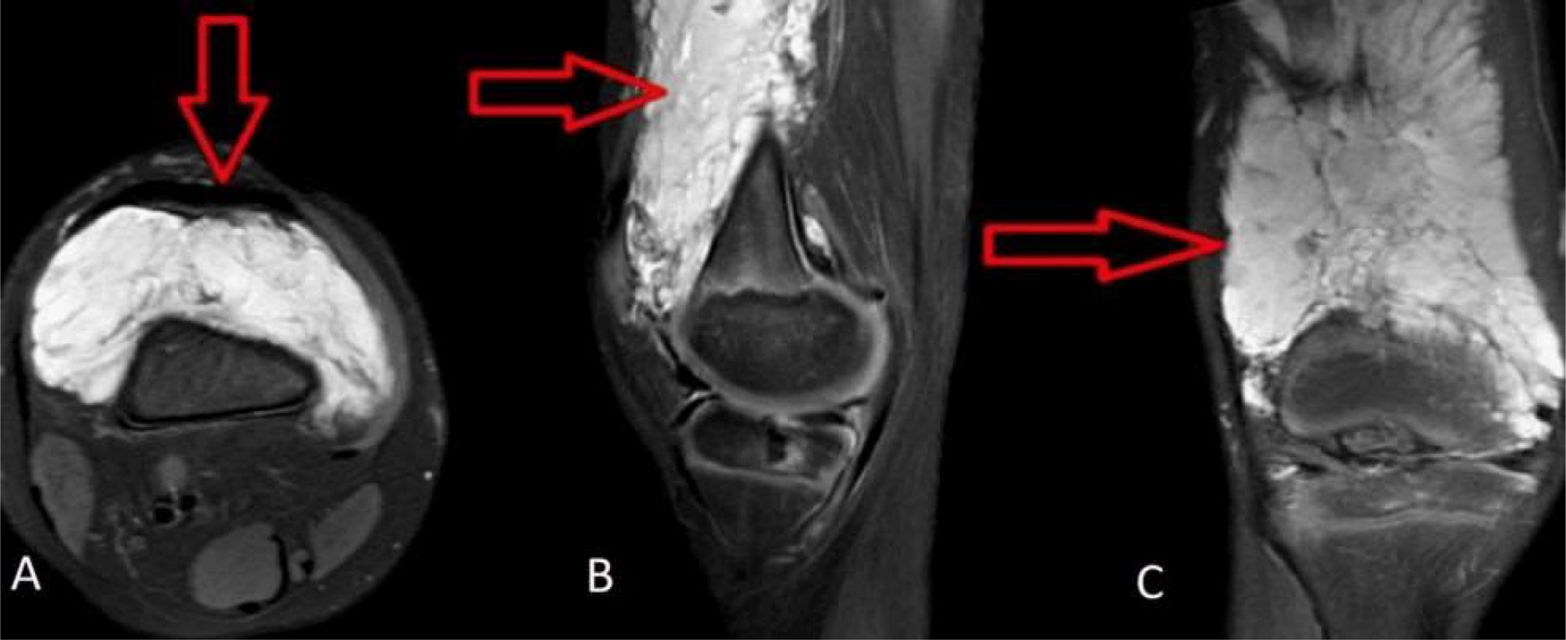
Magnetic Resonance Imaging (MRI) showing multiple, dilated, tortuous serpentine Proton Density Fat Saturated (PDFS) hyperintensity along vastus medialis muscle with hypertrophy of the muscle (red arrow). A-MRI PDFS axial section, B-MRI PDFS sagittal section C-MRI PDFS coronal section.

**Fig. 6:**
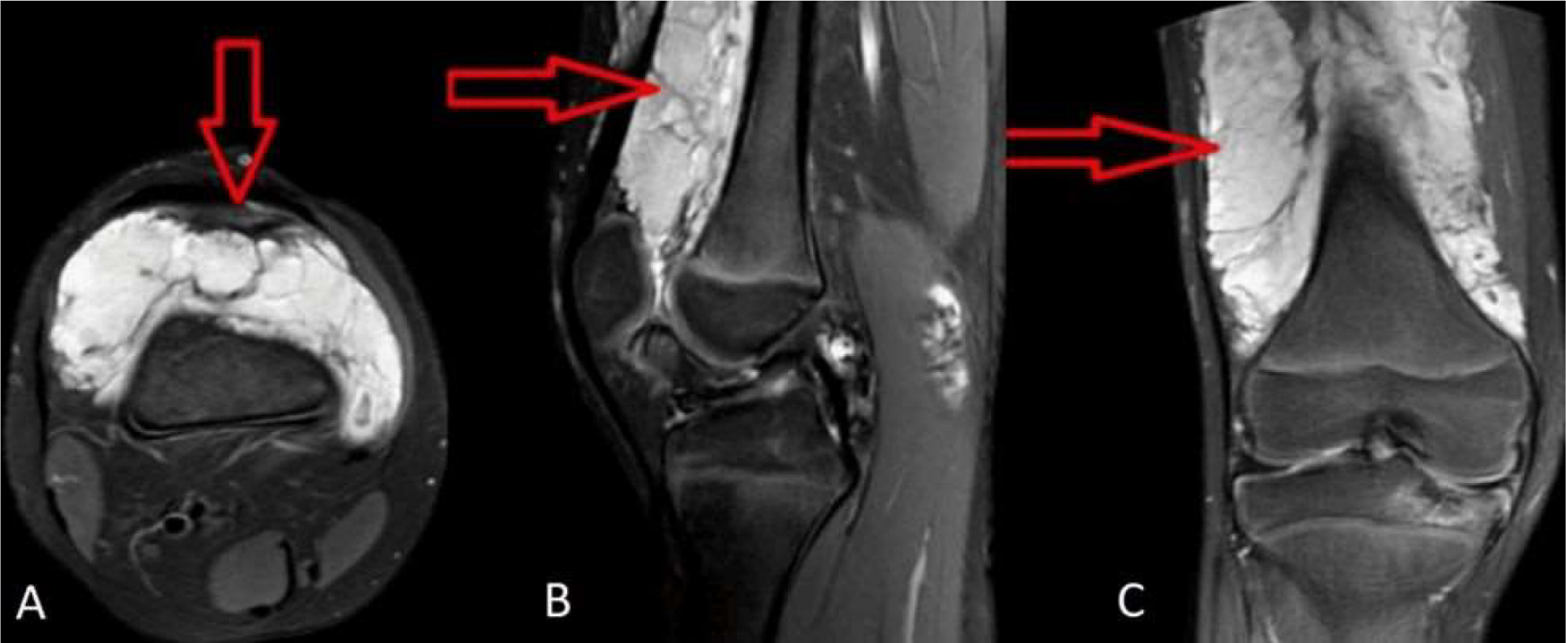
Magnetic Resonance (MRI) showing multiple, dilated, tortuous serpentine Proton Density Fat Saturated (PDFS) hyperintensity along vastus lateralis muscle with hypertrophy of the muscle (red arrow). A-MRI PDFS axial section, B-MRI PDFS sagittal section C-MRI PDFS coronal section.

**Fig 7:**
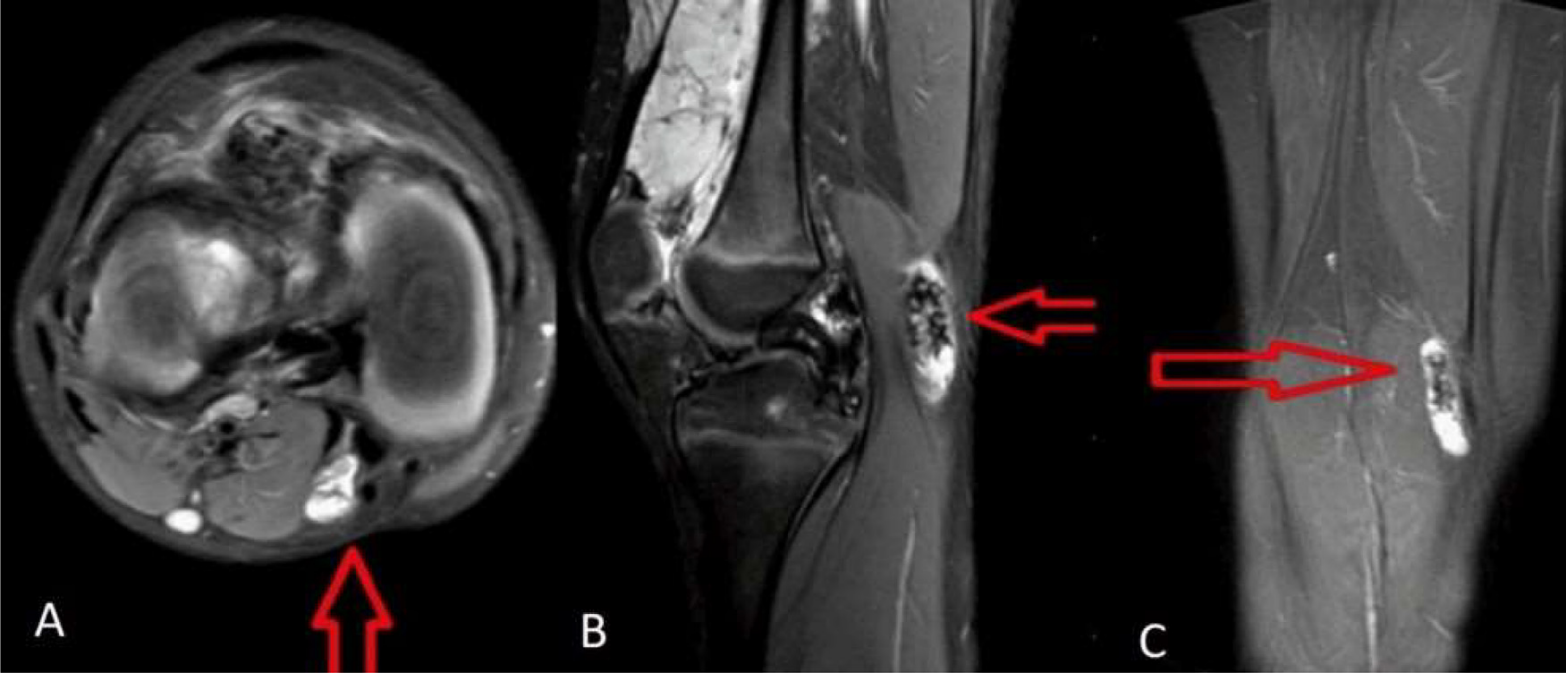
Magnetic Resonance (MRI) showing morrant bakers cyst in right popliteal fossa region (red arrow). A-MRI axial section, B-MRI sagittal section C-MRI coronal section.

Palpation did not elicit any tenderness. Systemic examination did not reveal any abnormalities. A plain X- ray of the right leg was performed in both the lateral and anteroposterior projections, but no significant findings were shown (Fig. 2). Further evaluation with ultrasound (USG) and colour flow imaging (CFI) revealed a multicystic anechoic mass with a few slow-flow vascular channels (Fig. 3). USG also showed showing well defined cystic lesion with internal echoes suggestive of morrant baker cyst in right popliteal fossa (Fig. 4). A multiplanar, multisequence MRI of the knee was performed which showed multiple heterogeneously enhancing dilated, tortuous, tubular, serpentine PD/STIR/T2 hyperintense signal vascular channels seen in the visualized portion of vastus medialis (Fig. 5), vastus lateralis (Fig. 5) and vastus intermedialis muscle, intermuscular plane and suprapatellar bursa region and fluid levels were seen in few of the lesions, in the inferior aspect of the thigh. Hypertrophy of the involved muscles was seen. There was no evidence any flow voids. The patient was diagnosed with VLM involving the vastus medialis, vastus lateralis, vastus intermedialis muscles, intermuscular plane, and supra-patellar bursa region, along with a Morrant Baker cyst (Fig. 6 and Fig. 7).

As the patient was asymptomatic, he was initially treated with non-steroidal anti-inflammatory drugs (NSAIDs) for pain management and compression therapy for edema control. During his subsequent follow up visit, successful pain control was achieved but swelling did not resolve. Hence, plan was made for sclerotherapy for the patient. With the assistance of ultrasound, a sclerosant (sodium tetradecyl sulfate) was administered into the malformation. The procedure was well tolerated, with no immediate complications noted. During the 3-month follow-up, the patient expressed a notable decrease in both pain and swelling. Gradual reintroduction of physical activity was advised, along with the recommendation for the patient to continue using a compression garment during sports activities. Subsequent biannual follow up was adviced.

## Discussion

Slow-flowing lesions called VLMs combine lymphatic and venous components. Before being renamed, they were referred to as lymphangio-hemangiomas or hemangio-lymphangiomas [1]. At birth, VLMs are present, increasing without ever involuting as the patient grows. The oral cavity, lip, temporalis, masseter muscle, tongue, and airway mucosa are frequent sites of involvement in the head and neck area, where about 50% of VLMs occur [2]. Rarely, they have also been noted in the lower extremity, mediastinum, gut and heart [3]. Hormonal influences, infection, trauma, or surgery caused by birth control pills, puberty, and pregnancy may cause VLMs to enlarge and become more symptomatic. While the lower limb is a very rare site of presentation for VLMs, it has been reported in some cases [4]. In our case, patient had history of knee trauma.

VLMs are a result of the vascular system developing abnormally during the embryonic stage due to disruptions in the late phases of angiogenesis (truncal stage) and the persistence of the AV anastomosis that existed during the embryonic stage. The TEK (chromosome 9p) gene, which codes for the endothelial cell tyrosine kinase receptor TIE2, mediates venous malformations through germline or somatic mutations. The elevated phosphorylation of TIE2 causes the usual recruitment of smooth muscle cells to become uncoupled from endothelial cells [5]. The receptor tyrosine kinase TIE2 gene mistakes have been connected to their existence.

VLMs are abnormal growths of dysplastic lymphatic and venous vessel structures [6]. These malformations can be categorized into slow flow, fast flow, or mixed types, with our case falling under the slow flow type. VLMs are made up of dilated lymphatic and venous channels as well as proteinaceous fluid and can either be congenital or acquired. The primary lymphatic system cannot communicate with the lymphatic channels. These channels have relatively little flow. Involvement in the lower limb is less frequent than in the craniofacial area. Vasculogenesis and angiogenesis are the two processes that shape the vascular system during development [1], with angiogenesis establishing connectivity between the peripheral and central circulation. The multi-cystic appearance of venolymphatic malformations is due to the dilated lymphatic channels, with larger veins, also present within the lesions. The presence of phleboliths is also a pointer towards venous malformations. While these lesions are commonly found in the head and neck regions, they can also occur in other parts of the body and can be congenital or acquired. There have been cases where spontaneous regression has been observed [7]. When examined, the lesions may have a heterogeneous nature and feel sponge-like or cystic. In the case discussed, the underlying deformity was diagrammatically presented alongside the lesion, which had a mixed consistency (Fig. 1). Although the skin on top may seem normal, a further infection could cause an ulcer to form [8].

Belov [9,10] developed a system for categorizing malformations based on their etiology and pathophysiology that was centered on the embryologic place of origin of the abnormality. There are two fundamental anatomical and pathologic types for each type of malformation: (A) truncular and (B) extra-truncular.

The classification of vascular abnormalities based on cellular properties, vascular flow parameters, and clinical behaviour was continuously updated and improved by the ISSVA. The categorization is still in use today thanks to the adoption of the final version in Rome in 1996 (Table 1) [11]. This system of classification comprises subcategories based on distinct traits, such as vascular tumors, vascular malformations, and other vascular anomalies. In general, the ISSVA classification has been essential in improving our knowledge of and approach to treating vascular abnormalities. Lymphatic malformations (LMs) and hemangiomas were classified separately from slow-flow venous malformations (VMs) and high-flow arteriovenous malformations (AVMs) (Table 2) [12].

**Table 1:** The classification systems for vascular abnormalities developed by the international society for the study of vascular abnormalities.

Vascular malformations		
Tumors	Simple	Combined
Hemangioma	Capillary (C)	Arteriovenous fistula (AVF)
Others	Lymphatic (L)	Arterio venous malformation
	Venous (V)	Cutaneous venous malformation
		Cutaneous lymphatic venous malformation
		Lymphatic venous malformation
		Cutaneous arterio-venous malformation
		Cutaneous lymphatic arterio-venous malformation

**Table 2:** Classification of vascular lesions in infants and children.

Hemangioma	Malformations
Proliferating phase	Capillary
Involuting phase	Venous
	Arterial
	Lymphatic
	Fistulae

Arteriovenous malformations and venolymphatic malformations exhibit distinct clinical behaviour and necessitate distinct diagnostic and treatment approaches. Venolymphatic anomalies are frequently asymptomatic, thus an accurate description of the location and size of the lesion can be obtained via an MRI scan. Depending on the size, location, and symptoms of the vascular malformation, there are several treatment options available, such as surgical excision, sclerotherapy, or embolization.

Venous abnormalities can be treated surgically, with sclerotherapy, with laser therapy, or with a combination of these methods [9]. Options for surgical intervention include electrocoagulation, irradiation, ligation, cryotherapy, or embolization [10]. Embolization is a minimally invasive surgical technique that, in some circumstances, can be used in place of surgery. To reduce blood loss during the surgery, a microcatheter is inserted to locate the feeder vessels before embolization.

Due to the vast range of clinical presentations that venous malformations might present with, they can be difficult to diagnose and manage. Since these lesions are uncommon, most doctors have little experience diagnosing and treating them, which can lead to incorrect diagnoses, insufficient care, high complication rates, and subpar patient outcomes.

## Conclusion

Rare vascular anomalies known as venolymphatic malformations can develop anywhere on the body. They can show as a soft tissue mass with or without symptoms, and a full radiological evaluation, such as an MRI and ultrasonography, can be used to confirm the diagnosis. Once it is established that the structural formation exists, appropriate care can lead to a full recovery. Venolymphatic abnormalities can be diagnosed and treated early to reduce the risk of consequences like bleeding, infection, and lymphedema. Surgical removal is the treatment of choice for most cases, but other options such as sclerotherapy, laser therapy, or a combination of these may be considered. Embolization can also be used as a minimally invasive surgical procedure in some cases. Follow-up should be done to rule out any recurrence. It is important for patients with venolymphatic malformations to be evaluated and managed at centers that have experience in treating vascular anomalies to ensure optimal outcomes.

VLM affecting the knee joint represents a rare phenomenon, presenting a challenge in both diagnosis and treatment. It is imperative to adopt a multidisciplinary strategy, engaging imaging experts, orthopedic practitioners, and interventional radiologists, in order to achieve precise diagnosis and effective management. Extra research endeavors and clinical case demonstrations are required to expand our knowledge of this atypical vascular irregularity and optimize therapeutic interventions

## Informed consent

Informed and written consent was obtained from the patient.

## Patient consent

Informed and written consent was obtained from the patient.

## Authors' contributions

SD and DN was involved in providing clinical details of the patient. PHP discussion on the pathology. GVM accumulated the results of the patient's radiological investigations. AK and RK was involved in collecting images and formatting data. All authors have read and approved the manuscript.

## Ethics approval and consent to participate

Written consent taken.

## Consent for publication

Written consent taken.

## Availability of data and material

N/A.
